# Over-expression of a poor prognostic marker in prostate cancer: AQP5 promotes cells growth and local invasion

**DOI:** 10.1186/1477-7819-12-284

**Published:** 2014-09-13

**Authors:** Jianping Li, Ziming Wang, Tie Chong, Haiwen Chen, Hechen Li, Gang Li, Xiaoqiang Zhai, Youfang Li

**Affiliations:** Department of Urology, the Second Affiliated Hospital of Medical College, Xi’an Jiaotong University, Xi’an, Shaanxi Province 710004 China

**Keywords:** Prostate cancer, AQP5, Prognosis, FISH, CTC

## Abstract

**Background:**

The aquaporins (AQPs), water channel proteins, are known playing a major role in transcellular and transepithelial water movement; they also exhibit several properties related to tumor development. The aim of the present study is to elucidate whether the expression of AQP5 is a strong prognostic biomarker for prostate cancer, and the potential role in the progression of prostate cancer cells.

**Methods:**

AQP5 expression was measured in 60 prostate cancer tissues and cells (both PC-3 and LNCaP) by immunohistochemistry and immunofluorescence assay. *AQP5* gene amplification was detected with FISH (fluorescence *in situ* hybridization). Proliferation and migration of cells and AQP5 siRNA cells were detected with MTT (3-(4,5-dimethylthiazol-2-yl)-2,5-diphenyl tetrazolium bromide) and Boyden chambers. Circulating tumor cells (CTCs) were detected by imFISH staining (CEP8-CD45-DAPI) assay.

**Results:**

The results showed that in 60 tumor specimens, 19 (31.7%) patients showed high level of AQP5 expression, while 30 (50.0%) showed a moderate, intermediate level of staining, and 11 (18.3%) showed an absence of AQP5 staining, respectively. High-expression of AQP5 protein frequently accompanied gene amplification detection with FISH. The AQP5 over-expression was also associated with TNM stage (*P* = 0.042), and lymph node metastasis (*P* = 0.001). The relationships between age or tumor size with the expression of AQP5 were not significant (*P* > 0.05). A positive correlation between the number of CTCs and AQP5 expression (*P* < 0.05) was demonstrated. In addition, patients who were negative for AQP5 had superior cumulative survival rate than those who were positive for it. Over-expression of AQP5 protein was also found in prostate cancer cells and cell proliferation and migration were significantly attenuated by AQP5-siRNA.

**Conclusions:**

We concluded that AQP5 in prostate cancer was an independent prognostic indicator. AQP5 over-expression was likely to play a role in cell growth and metastasis. These conclusions suggest that AQP5 may be an effective therapeutic target for prostate cancer.

## Background

Prostate cancer (PCa) is the second most frequent cause of cancer-related deaths among males in the USA [1]. Despite the widespread presence of clinically insignificant tumors in elderly men, PCa commonly has an aggressive phenotype that requires prompt intervention [2]. Therefore, it is extremely important to seek novel targets for therapeutic intervention.

Recently, studies have shown that tumor growth, development, invasion and metastasis depend on tumor microenvironment and tumor metabolism. Specifically, the water balance between the inside and outside of cells is essential to maintain the malignant epithelial cell function. Water can permeate the plasma membrane by simple diffusion, but bulk water flow is mediated by a channel mechanism through aquaporin (AQP) enzymes [3]. AQPs belong to a family of water-transporting proteins. Up to now, 13 members of the AQPs have been identified and can be subdivided into two groups on the basis of their permeability: AQP1, 2, 4, 5 and 8 are water-selective transporters, while AQP3, 7, 9 and 10, termed aquaglyceroporins, transport water as well as glycerol and other small solutes [4–6].

The role of AQPs in tumor pathogenesis has been identified. The study showed that aquaporin 3 was found to be over-expressed in malignant prostate tissue and may be involved in tumor progression [7, 8]. The expression of AQP1 is confined to prostate cancer cells and may be involved in microvascular alteration during tumor angiogenesis [9]. The over-expression of AQP5 was examined recently and showed that AQP5 is significantly associated with cervical cancer, ovarian cancer, and breast cancer progression [10]. It was shown that AQP5 plays a key role in cell proliferation. A recent study showed that AQP5 expression in colorectal cancer is significantly associated with lung metastasis that is possibly mediated by the activation of Ras, mitogen activated protein kinase (MAPK) and Rb signaling pathways [11]. However, the expression of AQP5 in prostate cancer and its clinical significance remain unexplored. To evaluate the potential of AQP5 as a novel prognostic marker of prostate cancer, we employed immunohistochemical, immunofluorescence, and FISH methods to detect the expression and amplification of AQP5 *gene* in clinical samples and prostate cancer cells, and immunofluorescence *in situ* hybridization (imFISH) staining to detect CTCs. We first analyzed the correlations between the expression of AQP5 and clinicopathologic features, CTCs, and prognosis of prostate cancer. Then, we also analyzed the effects of AQP5 knockdown on the cell proliferation and migration with 3-(4,5-dimethylthiazol-2-yl)-2,5-diphenyl tetrazolium bromide (MTT) and Boyden chambers.

## Methods

### Patient specimens

From January 2009 to June 2013, 60 patients were recruited from the Department of Urology Surgery, Second Affiliated Hospital of the Xi’an Jiaotong University. Samples were fixed with 4% formalin for histological studies. Of the 60 patients, median age at the time of surgery was 56.7 years (range 40 to 76 years). Tumor stage was recorded according to the classification of the International Union against Cancer (TNM stage). There were 7 patients with stage I tumors, 22 with stage II, 27 with stage III, and 4 with stage IV. All patients underwent operative surgical therapy, including partial resection or radical excision at the Department of Urinary Surgery, Second Affiliated Hospital of the Xi’an Jiaotong University. All patients were followed up and the median duration of follow-up was 23 months (4 to 52 months). All the studies were approved by the Human Subjects Committee of the Xi’an Jiaotong University, China. Written informed consent was obtained from the patient for the publication of this report and any accompanying images

### Cell culture

PC-3 and LNCaP prostate cancer cell lines (obtained from the American Tissue Type Collection, USA) were maintained in DMEM (GIBCO, Grand Island, NY, USA) and 10% heat-inactivated FBS and incubated in 5% CO_2_ humidified atmosphere at 37°C. Cells were grown to 80% confluency prior to treatment.

### Immunohistochemistry

AQP5 protein was detected immunohistochemically using a standardized streptavidin-peroxidase (SP) method. Tissue sections (4 μm) were incubated overnight with primary monoclonal antibody (1:100 dilution). Then the slides were incubated for 30 minutes with biotinylated goat anti-rabbit IgG (1:200 dilution). Color was developed using 0.02% 3,3’-diaminobenzidine (DAB) for 5 to 7 minutes. Negative controls for immunostaining replaced the primary antibody with nonimmune goat or rabbit serum. The staining was scored semiquantitatively as negative (0; no staining or < 10% staining), moderate (1; either diffuse weak staining or strong staining in less than 30% of cells per core), or strong (2; defined as strong staining of 30% or more of the cells). The antibodies against AQP5, and β-actin were purchased from Santa Cruz Biotechnology (Santa Cruz, CA, USA).

### Immunofluorescence assay

Tissue sections (4 μm) were incubated overnight. Slides were incubated with CY3-labeled anti-AQP5 (1:100) overnight. Fluorescent imaging was obtained with a confocal laser scanning microscope (Carl Zeiss MicroImaging, Inc., Thornwood, NY, USA). Exponentially growing cells were seeded on 25-mm square glass cover slips. Cells were fixed with 4% formaldehyde for 5 minutes, permeabilized with 0.2% solution of Triton X-100 in PBS, and blocked with 2% BSA-PBS for 30 minutes. Slides were incubated with CY3- or FITC-labeled anti-AQP5 (1:100) overnight, respectively. Cell nuclei were counterstained by 4’,6-diamidino-2-phenylindole (DAPI). Fluorescent imaging was obtained with a confocal laser scanning microscope (Carl Zeiss MicroImaging, Inc., Thornwood, NY, USA).

### Fluorescence *in situ*hybridization

*AQP5* gene amplification was detected with dual-color fluorescence *in situ* hybridization (FISH) using a Passvision AQP5 DNA probe kit (Vysis Inc. Downers Grove, IL, USA) according to its manufacturer’s instructions. Sections were baked overnight at 56°C, and were then deparaffinized followed by enzyme digestion and fixation. The slides were then denatured in 70% formamide/2 × standard saline citrate. After a buffer wash, 10 μl of a mixture of two directly labeled probes were added and hybridization was carried out at 37°C for 14 to 18 hours. The slides were then counterstained with DAPI, mounted, and stored in the dark before signal enumeration. AQP5-spectrum red probe contains a DNA sequence specific for the *AQP5* gene. CEP17 (chromosome enumeration probe 17)/spectrum green probe containing alpha-satellite DNA that hybridizes to the D17Z1 locus (centromere region of chromosome 17) was used as a control. Gene amplification was scored when a minimum of 20 cancer cell nuclei exhibited AQP5/CEP17 ratio ≥ 2, or when AQP5 signal cluster was observed.

### Cell viability assay

Cells were seeded (5 × 10^3^/well) in 96-well plates and cultured overnight. Then the MTT assay was used to determine cell viability. siRNA was added to the cells and further cultured for 24 hours. Then, MTT reagent (5 mg/ml) was added and incubation continued for an additional 4 hours. The reaction was terminated with 150 μl of dimethylsulfoxide (DMSO, Sigma-Aldrich, St Louis, MO, USA) per well. Absorbance values were determined using an MRX Revelation 96-well multiscanner (Dynex Technologies, Chantilly, VA, USA).

### RT-PCR (Reverse transcription-polymerase chain reaction)

Total RNA from cells was isolated using TRIzol reagent (GIBCO BRL, Grand Island, NY, USA). First-strand cDNA was synthesized from 2 μg of total RNA using the RevertAid Kit. The PCR primers that were used for the detection of AQP5 was synthesized as follows: 5’-CAG CTG GCA CTC TGC ATC TT-3’ (sense) and 5’-TGA ACC GAT TCA TGA CCA CC-3’ (antisense) and 5’-ATC GTG CGT GAC ATT AAG GAG AAG-3’ (sense) and 5’-AGG AAG AAG GCT GGA AGA GTG-3’ (antisense) for β-actin (179 bp). The PCR conditions were as follows: one cycle of denaturing at 94°C for 3 minutes, followed by 35 cycles at 94°C for 30 seconds, 55°C for 30 seconds, and 72°C for 35 seconds before a final extension at 72°C for 5 minutes.

### Western blotting

Briefly, 5 × 10^5^ cells were incubated on ice for 30 minutes in 0.5 ml of ice-cold whole-cell lysate buffer. The protein content of the cell was determined, and the cellular lysates were separated by 10% SDS-PAGE, and electro-transferred onto nitrocellulose membranes. After being blocked with 5% non-fat milk in tris-buffered saline and Tween (TBST), the membranes were incubated with primary antibodies at 4°C overnight, followed by secondary antibody for 2 hours. Immunoreactive bands were visualized using an enhanced chemiluminescence kit (Amersham Pharmacia Biotech, Piscataway, NJ, USA). The Western blot signals were quantitated by densitometric analysis using Total Lab Nonlinear Dynamic Image analysis software (Nonlinear, Durham, NC, USA).

### siRNA assay

To inhibit the expression of AQP5, we used siRNA oligonucleotide sequence: 5’-CGG UGG UCA UGA AUC GGU UTT-3’ (sense) and 5’-AAC CGA UUC AUG ACC ACC GCA-3’ (antisense). The oligonucleotides for negative siRNA were synthesized as sense 5’-UUC UCC GAA CGU GUC ACG UTT-3’, antisense 5’-ACG UGA CAC GUU CGG AGA ATT-3’. The PC-3 cells (2 × 10^6^) were transfected with siRNA targeted against AQP5 (100 nm/l) or a control siRNA (Qiagen, Valencia, CA, USA) using Lipofectamine 2000 (Invitrogen, Carlsbad, CA, USA). Cells were covered overnight before starvation.

### Cell invasion assay

Cell invasion assay was performed by employing Boyden chambers coated with 50 μg/ml of Matrigel solution. The cells were first seeded in 12-well plates at a concentration of 2.5 × 10^5^ per well and were cultured for 48 hours. The Matrigel invasion chamber was incubated for 24 hours in a humidified tissue culture incubator. After 24 hours, the noninvasive cells were removed from the upper surface of the separating membrane by gentle scrubbing with a cotton swab, and the invading cells were fixed in 100% methanol and stained with 0.1% crystal violet solution. They were then counted under a microscope at 200× magnification.

### Blood sampling and enrichment of circulating tumor cells

Seven and half milliliters of peripheral blood collected in BD Vacutainer tube (Becton, Dickinson and Company, Franklin, NJ, USA) were washed with PBS. All samples were collected after discarding the first 2 ml of blood. Red blood cells were removed. Resulting cell pellet was resuspended in PBS and subsequently incubated with 0.5 ml of antileukocyte surface marker CD45 monoclonal antibody coated magnetic beads for 30 minutes, followed by separation of magnetic beads using a magnetic stand (Promega, Madison, WI, USA). Supernatants were transferred into a new tube, and subsequently centrifuged at 800 g for 3 minutes. Cell pellets were spotted on glass slides, and then followed with imFISH (immuno- fluorescence *in situ* hybridization) staining.

### imFISH staining (CEP8-CD45-DAPI)

Tumor cells were negative enriched by immunomagnetic beads method, followed by identification with cytology analysis. FISH was performed using centromere DNA probes of chromosome 8 (yellow) (Vysis Inc. Downers Grove, IL, USA), and immunofluorescence assay was performed using anti-CD45 (red) (Santa Cruz Biotechnology Inc., Santa Cruz, CA, USA). The slides were washed three times with TBS. Cells were mounted with mounting medium containing the nuclear dye DAPI. A blinded review of the fluorescent images by three technicians confirmed the identity of the CTCs from 3-color fluorescent images that were magnified x400. Evaluation criteria for CTC identification from fluorescent images included both CEP8 ≥ 3 and CD45 (−) staining pattern overlying the DAPI staining of the nucleus.

### Statistical analysis and patient outcome

Data were analyzed by chi-square test (*χ*^2^) or two-sided Fisher’s exact test, as appropriate. Pearson correlation coefficient was used to measure the strength of the association among *AQP5* gene amplification. Survival rate was calculated by the Kaplan-Meier method, and differences were examined by the log-rank test. Factors found to be significant were then selected for a stepwise Cox’s multivariate proportional hazard model to determine their prognostic values. *P* < 0.05 was considered statistically significant. All statistical analyses were performed using SPSS Version 13.0 statistical software (SPSS, Chicago, IL, USA).

## Results

### Protein expression and gene amplification of AQP5 in prostate cancer

**Figure 1:**
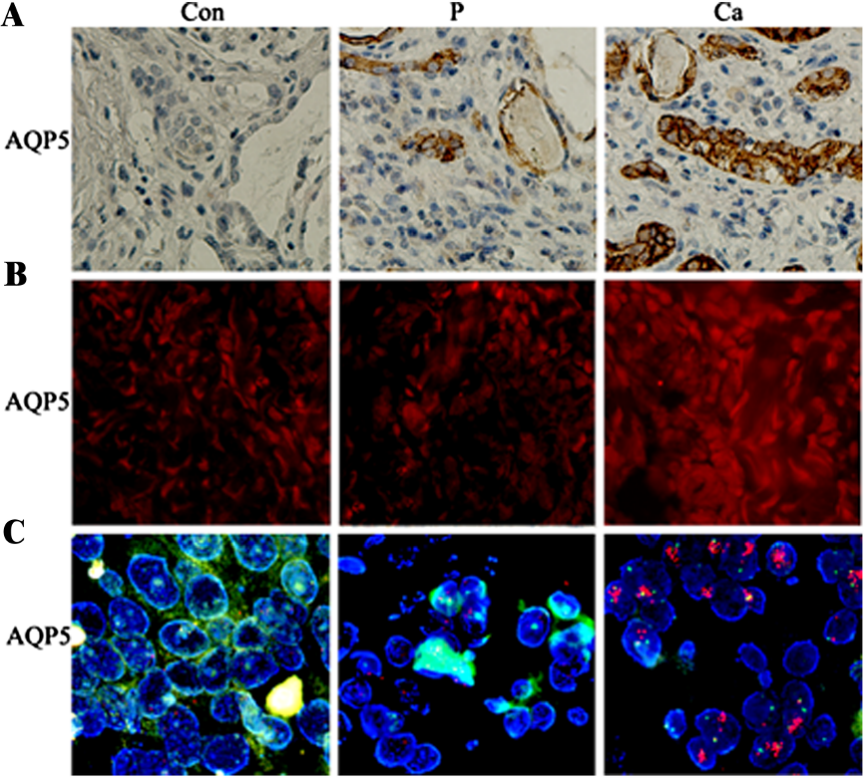
**Representative results of aquapore 5 (AQP5) expression by immunostain (A), immunofluorescence (B), and fluorescence** ***in situ*** **hybridization (FISH) (C).** **(A)** Normal tissue negative for AQP5 expression, paraneoplastic tissue negative for AQP5 expression, and tumor tissue with intense AQP5 expression. Paraffin sections were immunostained as described in the Methods section. **(B)** Immunofluorescence of AQP5 in prostate tissue: fluorescent imaging was obtained with a confocal laser scanning microscope. From top to bottom, AQP5 fluorescence signal in the cancer group, the control, and the paraneoplastic group. **(C)** *AQP5* gene amplification status was detected using FISH. *AQP5* gene in cancer group is amplified compared to the control and paraneoplastic group. Con: control, P: paraneoplastic, Ca: cancer.

To examine the expression status of AQP5 in prostate cancer, we first used immunohistochemical evaluation. The results highlight the difference observed in AQP5 immunostaining in the compartment (Figure 1A). In 19/60 (31.7%) patients, AQP5 expression was strong and in 30/60 (50.0%) patients AQP5 expression was moderate in the cancer group. AQP5 expression was only occasionally detected in paraneoplastic 11/60 (16.7%) and normal tissues 8/60 (13.3%).

To further determine AQP5 expression, prostate tissue was stained with fluorescence immunostaining and analyzed by confocal microscopy. The results showed that AQP5 fluorescence signal is substantially increased in the cancer group compared to the paraneoplastic and normal group (Figure 1B). The above results further suggest that AQP5 is over-expressed in prostate cancer.

Because FISH is a more accurate and sensitive assay (usually regarded as the gold standard) for detecting gene amplification compared to gene expression using immunohistochemistry, we further detected AQP5 amplification status using FISH. Interestingly, strong AQP5 expression had a positive correlation with *AQP5* gene amplification (r = 0.774, *P* = 0.009; Figure 1C; Table 1). In contrast, the expression of AQP5 was weak or absent where the *AQP5* gene was not amplified.

**Table 1:** **Relationship between protein expression of aquapore 5 (AQP5) and gene amplification**

		AQP5			
Gene	Case	0	1	2	***P*** -value
	n = 60	11 (%)	30 (%)	19 (%)	
AQP5					0.009
Amplification	34	0 (0.0)	17 (56.7)	17 (89.5)	
Normal	26	11 (100.0)	13 (43.3)	2 (10.5)	

### Relationships between AQP5 expression and clinicopathological parameters

Table 2 summarizes the associations of AQP5 protein expression and the clinicopathological parameters of prostate cancer. The strong expression of AQP5 was seen in 5 of 29 stage I to II cases (17.2%) and was significantly lower than that in stage III (37.0%, 10 of 27 cases) and stage IV (100.0%, 4 of 4 cases) cases (*P* < 0.05). AQP5 expression was also associated with lymph node metastasis (*P* = 0.001). We also investigated the relationship between age, tumor size with expression of AQP5, however, but no significant relationships were observed between these factors (*P* > 0.05).

**Table 2:** **Association between aquapore 5 (AQP5) protein expression and clinicopathologic factors in prostate cancers**

		AQP5			
Variables	Case	2	1	0	***P***-value
	n = 60	19 (%)	30 (%)	11 (%)	
Age (years)					0.785
> 60	36	13 (36.1)	16 (44.4)	7 (19.4)	
≤ 60	24	6 (37.5)	14 (58.3)	4 (16.7)	
Tumor size					0.929
≤ 2 cm	32	10 (31.3)	16 (50.0)	6 (18.8)	
> 2 cm	28	9 (32.1)	14 (50.0)	5 (17.9)	
Lymph node metastasis					0.001
Negative	22	2 (9.1)	10 (45.5)	10 (45.5)	
Positive	38	17 (44.7)	20 (52.6)	1 (2.6)	
ABCD stage					0.042
I/II	29	5 (17.2)	16 (55.2)	8 (27.6)	
III	27	10 (37.0)	14 (50.0)	3 (1.1)	
IV	4	4 (100.0)	0 (0.0)	0 (0.0)	

### Prognostic value of AQP5 in patients with prostate cancer

**Figure 2:**
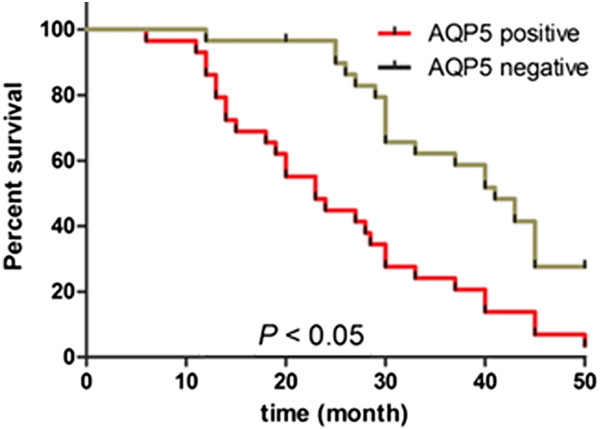
**Kaplan-Meier analysis of the overall postoperative survival curves in prostate cancer cases according to their immunohistochemical staining as positive or negative for aquapore 5 (AQP5).**

In order to determine the prognostic value of AQP5 for prostate cancer, we analyzed the cumulative survival of patients according to their AQP5 status (Figure 2). The AQP5-weak and non-staining specimens were merged as AQP5-negative, and strongly staining was regarded as AQP5-positive. The cumulative survival rate in AQP5-negative patients (n = 41) at 3 years was 64.3% (n = 26.4 median time 65.2 months). In contrast, the cumulative survival rate in AQP5-positive patients (n = 19) was 23.2% (n = 4.4 median time 20.7 months), a difference that is highly statistically significant (*P* < 0.05).

Multivariate analysis in our study revealed that lymph node metastasis (*P* = 0.048) and ABCD stage (*P* = 0.037) were the independent prognostic factors for overall survival time in patients with prostate cancer. Tumor diameter and other clinical parameters were also not independent prognostic factors.

### Expression of AQP5 correlates with CTCs enumeration

**Figure 3:**
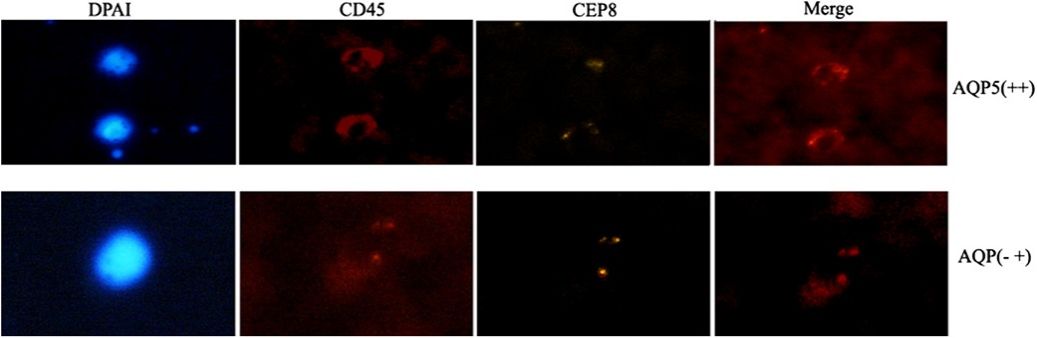
**Circulating tumor cells (CTCs) were stained with anti-CD45-CEP8-DAPI in peripheral blood of prostate cancer patients (×400).**

CTCs are shed from the primary tumor into the circulation. This has long been associated with metastasis and poor survival. In view of the obvious clinical relevance, CTCs have been recently recommended by the American Society of Clinical Oncology as an acceptable cancer marker. To further determine whether or not AQP5 is an adverse prognostic biomarker, the correlation between AQP5 expression and the number of CTCs was analyzed. CTCs were detected in 65.00% (13/20) of prostate cancer patients with strong AQP5 expression versus 30.00% (12/40) with low and absent AQP5 expression (*P* < 0.05; Figure 3).

### AQP5 expression in prostate cancer cells

**Figure 4:**
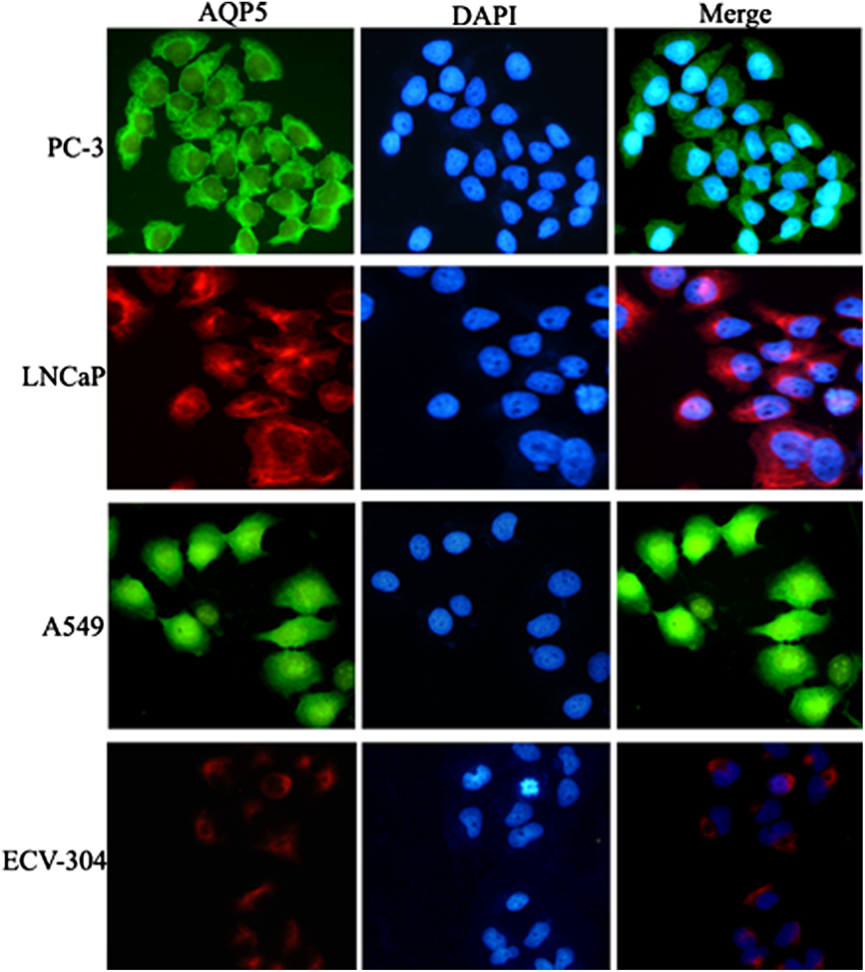
**Immunodetection of aquapore 5 (AQP5) proteins in PC-3 and LNCaP PCa (prostate cancer) cells.** The endothelial cell ECV-304 was used as a negative control and human lung cancer cell line A549 was used as a positive control for AQP5 expression. Fluorescent imaging was obtained with a confocal laser scanning microscope. AQP5 fluorescence signal in PC-3 is stronger than in the LNCaP cell.

To further determine AQP5 expression in prostate cancer cells, PC-3 and LNCaP cells were stained with fluorescence immunostaining and analyzed by confocal microscopy. The endothelial cell ECV-304 was used as a negative control and human lung cancer cell line A549, the AQP5-expressing cancer cell line was used as a positive control for AQP5 expression. Immunoblotting analysis revealed that both prostate cancer cell lines express AQP5 protein (Figure 4). AQP5 fluorescence signal in PC-3 is substantially stronger than LNCaP, so this cell was used for the further study.

### Proliferation and migration of PC-3 cells were inhibited in response to AQP5 silencing

**Figure 5:**
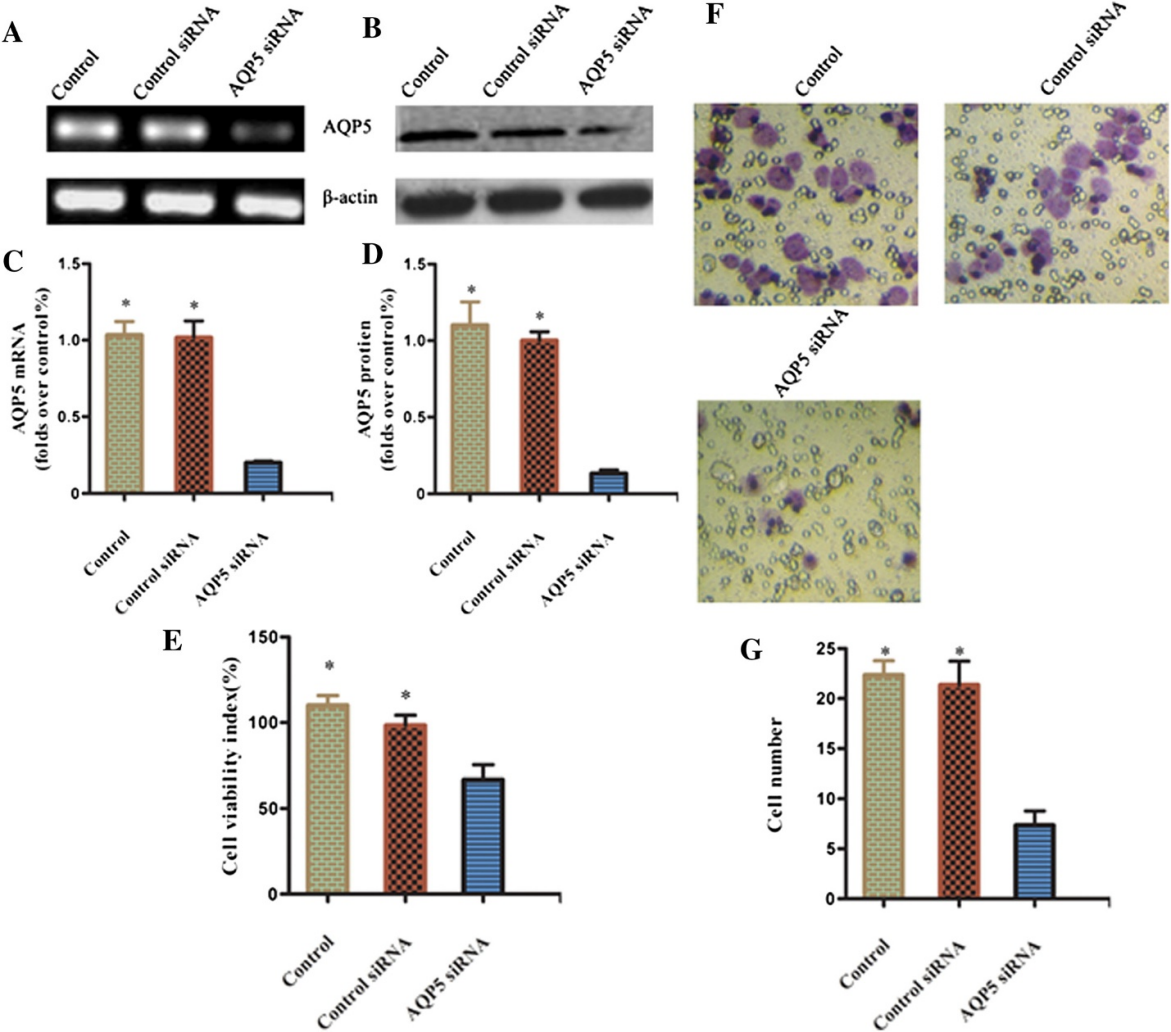
**Inhibition of cell proliferation and migration of PC-3 cell in response to aquapore 5 (AQP5) silencing. (A)** The efficacy of AQP5 siRNA for knockdown of AQP5 mRNA was confirmed by PCR. **(B)** The efficacy of AQP5 siRNA for knockdown of AQP5 protein was confirmed by Western blotting. **(C)** Quantification of mRNA. **(D)** Quantification of protein. **(E)** Effect of AQP5 silencing on the viability of PC-3 cells. **(F)** Effects of AQP5 silencing on the invasion of PC-3 cells. Data from at least three independent experiments with duplicate determinations are expressed as means ± SEM. *P < 0.05 was considered statistically significant. **(G)**: Columns in the graph are the diagram of the count analysis.

To examine whether AQP5 expression plays a role in the progression of prostate cancer, proliferation and migration of prostate cancer cells were examined in response to siRNA-mediated knockdown of AQP5. AQP5 mRNA and protein expression in PC-3 cell was significantly decreased by AQP5 siRNA transduction. The MTT result demonstrated that proliferation ability of PC-3 cell was significantly decreased with AQP5 silencing. Moreover, the number of migrated PC-3 cells was significantly decreased in response to AQP5 silencing (Figure 5).

## Discussion

Prostate cancer is one of the most aggressive and intractable malignant tumors, and its incidence and mortality have also rapidly increased in the United States [12]. Unfortunately, the mechanisms contributing to its carcinogenesis and progression are still poorly understood. In the present study, we aimed to determine the clinical significance of AQP5 expression in prostate cancer patients. AQP5, as a water channel protein, is also a predictor of early tumor recurrence, lymph node metastasis, and poor clinical outcome in cancer patients [13]. Our results revealed that AQP5 is up-regulated in prostate cancer and this expression is accompanied by AQP5 DNA amplification. AQP5 protein was also expressed in prostate cancer cell lines, PC-3 and LNCaP. Our results also suggested that AQP5 is closely correlated with advanced TNM stage, CTCs, and poor prognosis. Cell culture experiments also revealed the role of AQP5 in tumor cell proliferation and migration. These findings extend our understanding of AQP5 role as a negative prognostic marker.

Aquaporins (AQPs) are a family of small membrane transport proteins [14]. Compelling new evidence has implicated several members of the AQP family, including AQP5, in tumorigenesis. Moreover, intensive studies have been focused on the modulation of AQP5 expression in tumor cells and its effects on tumorigenesis, tumor growth and progression [15–18]. AQP5, a 21 to 24 kDa protein, is a component of caveolae, invaginated microdomains of the plasma membrane. AQP5 was initially described as the main structural protein in caveolae and was believed to be a key molecule involved in oncogenic transformation and malignant progression [11]. AQP5 plays an important regulatory role in several signaling pathways in cellular transformation, including those mediated by the Src family of tyrosine kinases, epidermal growth factor receptor, protein kinase C, Wnt, and Erk1/2 [10]. AQP5 has been suggested to act either as a tumor suppressor or as an oncogene, depending on the tumor type. These various effects may be explained by the activation status of different domains of AQP5 or the expression levels of other molecules that interact with AQP5 in these different signaling pathways [19]. Recently, Lee *et al.* reported that AQP5 over-expression was significantly associated with lymph node involvement and a poorer prognosis in patients with breast cancer [13]. Huang *et al.* also verified that AQP5 promoted the proliferation and migration of human gastric carcinoma cells. Interestingly [20], Fischer *et al.* performed differential display to find that AQP5 was expressed only in normal colonic tissue and not, or to a lesser extent, in cancer cells [21]. The clinical significance of AQP5 in prostate cancer remains largely unexplored. Our results showed that AQP5 was mainly expressed in prostate cancer cells. The expression patterns of AQP5 in prostate tissues that we detected are consistent with the results of previous studies [6, 10, 11, 22]. Furthermore, we determined that high levels of expression of AQP5 were correlated with gene amplification in our limited cases study, which is the first reported in prostate cancer. In addition, in the present study, we also demonstrated significantly decreased cell proliferation and migration of prostate cancer cells with AQP5 silencing. Osmolar changes have been revealed to affect cell proliferation, as previously demonstrated by the negative effect of hypertonicity on cell proliferation [23]. Tumor cells bind to the basal membrane surface, and the formation of pseudopodial protrusions at the leading edge of migrating cells is the earliest step in locomotion. Studies have suggested that AQP-facilitated water permeability in cell protrusions enhances their formation and thus the rate of cell migration. AQP expression and tumor cell water permeability are potentially important determinants for tumor spread and metastasis [24–26]. However, the possibility that AQP5 may promote cancer cell migration by other mechanisms could not be excluded. Further studies are required to elucidate the specific underlying molecular mechanisms.

CTCs are tumor cells shed from the primary tumor into the circulation. The presence of CTCs in the peripheral blood of patients has long been associated with metastasis and poor survival and is now considered an acceptable cancer marker. Yet, current techniques are limited [27, 28]. The only commercially available CTC test (CellSearch; Veridex LLC, North Raritan, NJ, USA) has a detection rate of 50% in late-stage patients [29, 30]. In the current study, we detected CTCs harboring negative enrichment using an immunomagnetic beads method, followed by identification with cytologic analysis, immunofluorescence, and imFISH. This combination of methods resulted in detection rates up to 65.00% in patients with AQP5 over-expression, suggesting that imFISH staining could be used as a detection method for CTCs in future studies. In addition, our study verified that AQP5 over-expression is associated with the likelihood of metastasis compared to lower expression of AQP5.

## Conclusions

The AQP5 protein is up-regulated in prostate cancer and is closely related to advanced ABCD stage, lymph node metastasis, CTCs and poor prognosis. Moreover, AQP5 expression was associated with cell proliferation and migration. Although the mechanisms require further elucidation, AQP5 might be used as a novel biomarker for prostate cancer aggressiveness.

## Abbreviations

AQPs: the aquaporins
bp: base pairs
BSA: bovine serum albumin
CTCs: circulating tumor cells
DAB: diaminobenzidine
DAPI: 4’,6-diamidino-2-phenylindole
DMEM: Dulbecco’s modified Eagle’s medium
DMSO: dimethylsulfoxide
FISH: fluorescence *in situ* hybridization
imFISH: immunofluorescence *in situ* hybridization
MAPK: mitogen activated protein kinase
MTT: 3-(4,5-dimethylthiazol-2-yl)-2,5-diphenyl tetrazolium bromide
PBS: phosphate-buffered saline
PCa: prostate cancer
RT-PCR: reverse transcription-polymerase chain reaction
SP: streptavidin-peroxidase
TBST: tris-buffered saline and Tween.
